# Testosterone Depletion Induces Demethylation of Murine Reelin Promoter CpG Dinucleotides: A Preliminary Study

**DOI:** 10.1155/2015/286369

**Published:** 2015-10-07

**Authors:** Victor Augusto Moraes da Silva, Marília de Souza Dantas, Leonardo Agostinho de Castro Silva, Juliana Garcia Carneiro, Bruno Luiz Fonseca Schamber-Reis

**Affiliations:** Department of Medical Genetics, School of Medical Sciences, CESED/FCM, Avenida Senador Argemiro De Figueiredo 1901, Itararé, 58411-020 Campina Grande, PB, Brazil

## Abstract

Schizophrenia (SZ) is a debilitating mental disorder characterized by psychotic events, abnormal social behavior, false beliefs, and auditory hallucinations. Hypermethylation of the promoter region of reelin (*RELN*), a gene involved in regulation of neuronal positioning during telencephalic development, is strongly associated with low protein expression in several cortical structures and promoter hypermethylation in brain from postmortem SZ subjects. Recent experimental data suggests that testosterone is able to promote *RELN* demethylation, although no direct evidence of hormonal influence on reelin promoter methylation was obtained. We investigated if reduced levels of plasma testosterone in adult male mice lead to *Reln* promoter demethylation. Animals were administered with flutamide, an antiandrogenic compound, and reelin promoter methylation was assessed using methylationspecific PCR using bisulfite DNA from cerebellum. We found that flutamide was able to significantly lower plasma testosterone when compared to control mice, and treatment did not influence animal survival and body weight. We also show that low plasma testosterone was associated with demethylation of a cytosine residue located at −860 in reelin promoter region. These preliminary data suggest that androgenic hormones can influence cerebral reelin demethylation. To our knowledge, this is the first experimental approach directly linking testosterone depletion and *RELN* promoter methylation.

## 1. Introduction

Schizophrenia (SZ) is amongst the top ten causes of health burden in the world affecting about 1% of population [1]. Patients show cognitive, motor, and social impairment early on childhood; other symptoms like anxiety and depression can emerge during adolescence, with social withdrawal, prodromal symptoms, and psychotic episodes being characteristic at early adulthood [2]. Although the etiology of SZ is not fully understood, the neurodevelopmental hypothesis proposes that SZ symptoms result from the interplay between stress-inducing factors during pregnancy (e.g., maternal stress, prenatal exposure to viral infections and inflammation, fetal hypoxia, and low birth weight) and childhood (e.g., child abuse, migration, and urbanicity) [3]. Pharmacological treatment is based on administration of antipsychotics, which confer palliative effects limited to controlling psychosis events and dependent on side effects [4].

The clinical aspects of SZ seem to be related to damage in neocortical and cerebellar areas of SZ patients, affecting Purkinje cells morphology and distribution [5, 6]. Several genes including reelin (*RELN*), glutamic acid decarboxylase 67 (GAD67), and disabled-1 (DAB-1) are strongly believed to be involved in the pathophysiology of SZ. Reelin is an extracellular matrix-associated glycoprotein expressed in the developing brain by Cajal-Retzius cells in cortex and hippocampus and by cerebellar granule cells in the external granule cell layer [7–9]. Reelin binds to lipoprotein receptors and signals for Dab1 phosphorylation [10], playing an important role during brain development in controlling neuronal migration processes, synaptogenesis, axonal branching, and long-lasting maintenance of synaptic function in adults [8, 11–14]. Although reelin is believed to be involved in SZ development, it has been also linked to other neurodevelopmental disorders as autism, bipolar disorder, major depression, and Alzheimer's disease, as they share abnormal reelin expression in the brain [15].

Concordant studies showed that expressions of reelinmRNA and protein were reduced by around 50% in several cortical structures of postmortem brain from SZ patients when compared to the same corresponding areas in nonpsychiatric subjects [13, 16]. Several works reported that reelin deficiency was correlated with the intensity of upstream RELN CpG island promoters methylation [8, 17–19]. DNA methylation is an epigenetic modification characterized by 5-methylation of cytosine (5mC) restricted to gene promoter CpG dinucleotides. This process is catalyzed by DNA methyltransferases (DNMTs), which favors transcriptional repression by recruiting methyl-CpG binding domain (MBD) proteins that attract histone deacetylases (HDACs) and corepressors that induce chromatin remodeling [20]. Indeed, it was shown that in SZ there is an exacerbated expression of DNA methyltransferase 1 (DNMT1) [21, 22]. Administration of L-methionine, a precursor of S-adenosylmethionine involved in DNA methylation providing methyl groups, can exacerbate symptoms in most SZ patients and can model SZ molecular neuropathologies in mice [9, 17].

It is well accepted that* RELN* promoter hypermethylation is associated with reelin hypoactivity in SZ patients. Some drugs, like valproate and doxorubicin, can alleviate cognitive deficits and other symptoms observed in SZ and bipolar disorder by inhibiting DNMTs and HDACs and increasing the levels of acetylated histones, leading to an upregulation of reelin expression in a dose-dependent manner [9, 23, 24]. However, drugs that act as DNMT inhibitors are expected to lead to an upregulation of several other genes and potential side effects, which still pose a disadvantage in using this class of molecules for treating SZ patients [25].

Insufficient knowledge exists about how environmental agents can lead to gene demethylation. Many proteins, drugs, and hormones can induce pathological gene methylation that increase disease susceptibility [26]. Sex hormones such as prolactin, estradiol, and estrogen signal for promoter methylation since the target gene is responsive to environmental influence [27]. However, scarce data are available in the literature about the way the hormone testosterone is controlling reelin methylation. In humans, indirect evidence showed that cerebral reelin expression was shown to be higher in women compared to men [28]. In addition, methylation of* RELN* promoter in postmortem temporocortical samples from prepuberal normal individuals was scarce, while postpuberal samples were highly methylated [29]. A more direct evidence of testosterone influence on reelin expression was obtained by administering exogenous testosterone to male European starlings, which promoted a significant reduction of cerebral reelin expression [30], although no data about* RELN* promoter methylation after treatment was obtained.

In this work we tested our hypothesis that testosterone leads to* Reln* promoter methylation in mice. We found that adult male mice treated with flutamide, an antiandrogenic compound [31–33], were able to lower plasma testosterone, which was correlated with reelin promoter CpG demethylation. To our knowledge, this is the first experimental approach directly linking testosterone depletion and modulation of reelin promoter methylation.

## 2. Material and Methods

### 2.1. Animals and Drug Administration

We used adult male* Swiss* albino mice, with age between 8 and 10 weeks and 30–35 g body weight. Animals comprised in experimental group (*n* = 5) were IP injected with 50 *μ*g of flutamide (2-methyl-*N*-[4-nitro-3-(trifluoromethyl)phenyl]-propanamide, Sigma-Aldrich, EUA) per gram of body weight diluted in 50 *μ*L of DMSO 100% (Vetec, Brazil). Mice from control group were administered with vehicle only. Treatment occurred once a day, for a period of 30 days. After flutamide treatment, mice were euthanized and had their cerebellum harvested. Brain tissue was immersed in* RNAlater* solution (Ambion, USA) and stored at −80°C.

### 2.2. Plasma Testosterone Dosage

Serum was diluted 1 : 20 in PBS 1x and total plasma testosterone was quantified using an Immulite 2000 Total Testosterone automated assay system (DPC, USA), according to manufacturer's recommendations. This method involves a competitive immunoassay based on ligand-labeled testosterone and a polyclonal antibody specific for testosterone. Quantification was performed using samples from five mice per group and results were expressed as nanograms of testosterone per microliter of plasma.

### 2.3. DNA Extraction

Whole cerebella were let to defrost on ice, and a total of 25 mg of tissue was washed with saline 0.8%. Samples were initially disrupted with a 5 mL syringe and washed again with saline, and pellet was submitted to genomic DNA extraction using HiPurA Multi-Sample DNA Purification Kit (Himedia, India) according to manufacturer's protocol. DNA obtained was quantified using Nanovue Plus (GE Healthcare, EUA) and diluted in TE buffer for long-term storage.

### 2.4. Methylation Specific PCR Primer Design

**Figure 1:**
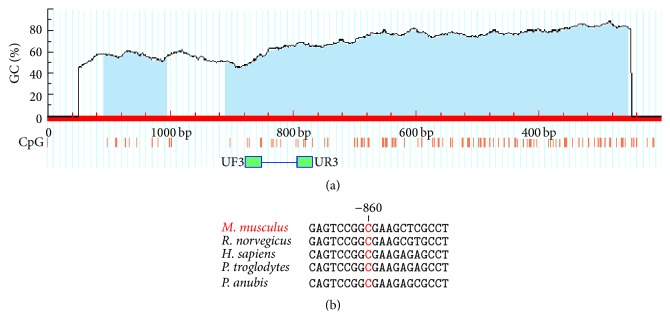
(a) Murine* Reln* CpG island prediction and MSP primer design. Blue regions delimited on mouse reelin gene promoter sequence (first 1200 bases) indicate CpG islands predicted by MethPrimer software (http://www.urogene.org/methprimer/). General parameters for CG-rich regions are depicted in Section 2.4. The small red lines indicate C residues from CpG dinucleotides alongside Reln promoter region. Green boxes indicate annealing regions for MSP forward (UF3) and reverse (UR3) primers. (b) Alignment of segments of DNA sequences from reelin promoter regions of different species showing the conserved cytosine at position −860.

To assess Reln gene promoter methylation, we adopted methylation specific PCR (MSP) to discriminate between methylated and unmethylated DNA [34]. This technique involves primers capable of discriminating the unmethylated version of specific C residue from its methylated counterpart. Mouse reelin promoter sequence (RefSeq NM_011261, MGI accession number 103022) was delimited in this work as −1200 bases before start codon to select for CpG dinucleotides at CG-rich regions (CpG islands) using MethPrimer software [35], at which results are depicted in Figure 1(a). Criteria used for CpG island prediction were window size > 100, window shift of 1 pb, GC percent > 50, and observed/expected CpG ratio of 0.6. Once CpG island was delimited, we obtained suitable primer pairs to distinguish between unmethylated and methylated states of a cytosine residue located at position −860 from initial ATG. This residue is highly conserved amongst primates and rodents (see Figure 1(b)) and chosen to be analyzed by MSP. Primers used for MSP were as follows: unmethylated DNA specific: forward, 5′-TTT TTA GTA ATG TGT AAA TAT AGA GTT TGG-3′; reverse, 5′-AAA TAA TAC AAA ACC AAA TCA TCA AA-3′; methylated DNA specific: forward, 5′-TTA GTA ACG CGT AAA TAT AGA GTT CG-3′; reverse, 5′-ATA ATA CGA AAC CAA ATC GTC GA-3′. Expected PCR product sizes for both primer pairs are 105 bp and 100 bp, respectively. Both sense primers were expected to anneal its second 3′ base within the C residue to be tested in order to maximize stringency of detection.

### 2.5. Bisulfite Modification

Modification of DNA by sodium bisulfite was conducted following previous protocol published elsewhere [36]. Briefly, DNA (1 *μ*g) in a volume of 25 *μ*L was added to 2.75 *μ*L of NaOH 2 M and incubated for 10 min at 37°C. Fifteen microliters of hydroquinone 10 mM (Sigma, EUA) was added followed by addition of 260 *μ*L of sodium bisulfite 3.6 M (Sigma), both freshly prepared. After vortexing, the mixture was layered with mineral oil and incubated for 16 hr at 54°C on water bath. Bisulfite modified DNA was purified using HiPurA Genomic DNA Miniprep Purification Kit (Himedia, India) according to manufacturer and eluted into 200 *μ*L of water. Modification was completed by NaOH treatment (final concentration 0.3 M) for 5 min at room temperature, followed by addition of 24 *μ*L of sodium acetate 3 M, pH 5.2, followed by ethanol precipitation. Pelleted DNA was resuspended in 50 *μ*L of TE buffer and stored at −20°C.

### 2.6. MSP/PCR Setup and Cycling Conditions

PCR reactions were carried out in a final volume of 25 *μ*L, containing 12.5 *μ*L of GoTaq Green Master Mix (Promega, EUA), 5 pmol each of the methylated or unmethylated primers, and 50 ng of bisulfite modified genomic DNA from treated and untreated mice. Bisulfite modified* in vitro* methylated mouse DNA and double-stranded DNA were used as positive and negative controls, respectively. PCR cycling conditions consisted of an initial denaturation step at 95°C for 5 min, followed by 40 cycles of 95°C for 45 sec, 58°C for 45 sec, and 72°C for 45 sec, finishing with a final extension step at 72°C for 5 min. Two microliters of each PCR product was analyzed by electrophoresis on a nondenaturing 0.8% agarose gel stained with ethidium bromide and directly visualized under UV light illumination using a L-PIX image analyzer (Loccus Biotecnologia, Brazil). A semiquantitative end-point analysis of band intensities was performed using Fiji image processing package [37].

## 3. Results

**Figure 2:**
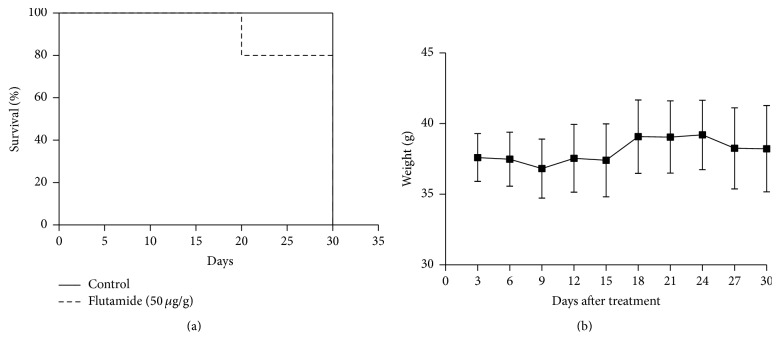
(a) Kaplan-Meier survival curves from control (full line) and flutamide-treated mice (dashed line). The *x*-axis shows days after treatment; the *y*-axis shows proportion of mice that survived. Animals were euthanized at day 30. Differences in survival were measured by log-rank (Mantel-Cox) test (*p* = 0.453). (b) Mean body weight of flutamide-treated mice. Measurements were done every three days until day 30. Values represent median ± SEM (*n* = 5). Data is representative of three independent experiments.

We were interested in verifying the effects of flutamide treatment on mice survival. Animals were daily treated with 50 *μ*g of the compound diluted in DMSO 100% per gram of body weight, during 30 days. Flutamide administration allowed four out of five mice from treated group to survive until day 30, although eventually one mouse died possibly of opportunistic infections after recurrent intraperitoneal administration (Figure 2(a)). We also found that IP administration of 50 *μ*L of DMSO 100% was not toxic to mice, as all mice were able to survive until the end of the experiment. In addition, we evaluated the body weight of control and treated mice every three days, for 30 days. We found that body weight did not vary significantly during this period (Figure 2(b)), suggesting amenable effects of flutamide on body weight at the dose used.

**Figure 3:**
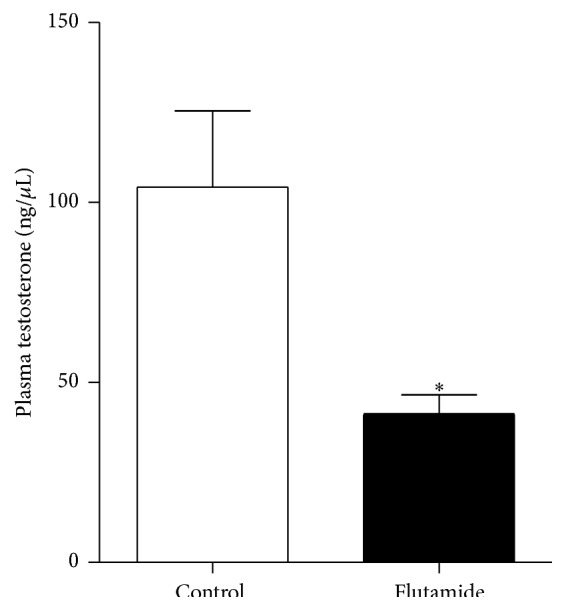
Total plasma testosterone of vehicle and flutamide-treated mice. Values represent median ± SEM (*n* = 5 per group). Difference was considered significant (*p* = 0.035, Mann Whitney exact test). Data is representative of three independent experiments.

Experimental data showed that flutamide causes a short-term increase in plasma testosterone in humans, followed by a decrease in its concentration. However, to our knowledge, no previous data regarding the effects of flutamide on plasma testosterone levels from healthy mice was obtained. Mice were euthanized after 30 days of flutamide administration and plasma testosterone was quantified. We found that mice treated with flutamide showed lower plasma levels of testosterone compared to control group treated with vehicle only (Figure 3).

**Figure 4:**
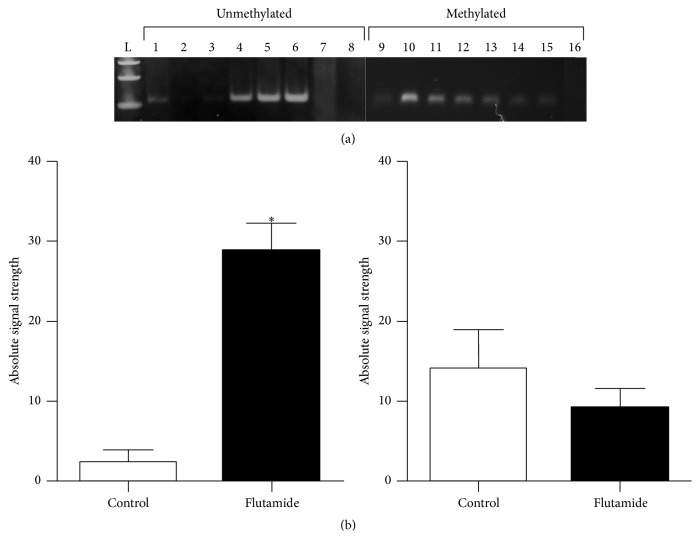
Methylation specific PCR evaluation of reelin promoter after flutamide administration. (a) MSP analysis of CpG cytosines located at position −860 of mouse reelin promoter. Gel images show results obtained from control mice (lanes 1–3 and 9–11) and flutamide-treated animals (lanes 4–6 and 12–14) using specific primers designed to detect unmethylated or methylated cytosines.* In vitro* methylated mouse DNA (lanes 7 and 15) serves as positive and negative controls for each MSP primer, respectively. Genomic double-stranded DNA (lanes 8 and 16) was used as additional control to show that MSP primers do not amplify unmodified DNA. L, 100 bp DNA ladder. (b) Semiquantitative end-point comparison of band intensity was carried out using Fiji Software; *n* = 5 mice per group. Values represent median ± SEM; ^*∗*^ *p* < 0.05.

We also determined to evaluate possible alterations in the methylation status of a specific cytosine residue in the Reln promoter region after testosterone depletion in mice. By adopting MSP technique, we designed a sense primer that anneals specifically to the unmethylated version of the C residue located at position −860 and another one that anneals to its methylated counterpart. After sample electrophoresis of MSP reactions using bisulfited DNA as template, we obtained stronger PCR products using the unmethylated primer (UF3) for flutamide-treated mice DNA and faint bands correspondent to control samples (Figure 4(a)). On the other hand, when using methylated primer (MF3), we observed an overall similar profile of intensities between control and flutamide-treated mice, although with a discrete increase in intensity for control mice. We quantified band intensities using an image processing software. Our results showed significant differences between groups only when testing for unmethylated C, suggesting demethylation of this residue after flutamide treatment and testosterone depletion (Figure 4(b)).

## 4. Discussion

Several works linked schizophrenia to an imbalance of reelin,* Gad67,* and* Dab-1* expression. Improper reelin production was demonstrated to be strongly associated with disease etiology and progression. The most consolidated experimental data is that postmortem SZ patients show higher reelin promoter methylation, which leads to low protein expression in neurons compared to healthy subjects [8, 18]. In this scenario, literature presents sufficient molecular data about reelin expression and promoter methylation in highly symptomatic patients diagnosed for SZ. However, a very small number of studies aimed to elucidate whether hormonal changes could signal and control reelin gene expression. Cerebral reelin expression in women was demonstrated to be higher when compared to men [28] and lower reelin promoter methylation in postmortem temporocortical samples from prepuberal normal male individuals (nonpsychiatric subjects) was also shown [29]. Direct experiments involving testosterone administration to male starlings showed that the treatment was able to lower cerebral reelin protein expression [30]. Nevertheless, these works did not collect molecular data regarding possible changes in reelin promoter methylation status. Thus, insufficient data about how testosterone could influence reelin protein methylation lead us to investigate whether testosterone influences* RELN *promoter methylation. By treating male mice with flutamide in a long-term period, we were able to verify that plasma testosterone levels dropped, compared to nontreated animals, and this was followed by CpG demethylation of reelin promoter.

We showed that flutamide administered to healthy mice was able to decrease plasma testosterone concentration compared to control animals, which received only DMSO. Flutamide is an antiandrogenic compound that disrupts the development of male androgen-dependent tissues, leading to abnormalities in the male reproductive system as hypospadias and reduced anogenital distance [38]. Flutamide has been also described to attenuate heat-induced multiple organ damage and lethality in mice, the same effect observed in mice that had most of plasma testosterone depleted by castration [39]. In humans, flutamide has been used for prevention and treatment of prostatic cancer [32, 33, 40]. Our experimental data showed that flutamide administration alone leads to a decrease in plasmatic testosterone. Experimental data previously published elsewhere showed that flutamide could suppress testicular testosterone production, for example, when administered together with Zoladex, a gonadotrophin hormone releasing hormone (GnRH) [31]. We observed a decrease in plasma testosterone levels after 30 days of administration of flutamide diluted in pure DMSO to mice. We hypothesize that the interaction of flutamide with DMSO also favored testosterone depletion, although this effect needs to be further clarified.

We also showed, by performing MSP analysis, that depletion of plasmatic testosterone in male mice is associated with demethylation of a cytosine residue at position −860 located at reelin promoter region. In our experiments, we observed a high PCR amplification corresponding to the annealing of the primer specific for unmethylated cytosine in DNA samples from flutamide-treated mice. Significant differences were not observed between groups when testing for the presence of methylated cytosine, where overall similar intensities were detected. Considering that MSP technique is markedly more sensitive than Southern analysis, allowing detection of small numbers of methylated alleles [34], we believe that flutamide administration promoted partial demethylation of reelin alleles in cerebral tissue, as we could also detect methylated alleles. In addition, several works involving promoter hypermethylation analysis using DNA extracted from tumor samples showed that MSP detection of both methylated and unmethylated alleles in the same sample was possible, yielding bands with similar intensities [8, 41, 42]. We can exclude the possibility of cross-contamination, as extreme care was taken during tissue harvesting and samples handling, positive and negative controls were used, and concordant results were obtained from independent experiments. In addition, sharp differences between groups were observed when using the sense primer for the unmethylated residue, which would not be possible if cross-contamination had occurred during sample manipulation. Thus, it is reasonable to consider that reelin methylated and unmethylated alleles coexist in cerebellum, and another level of regulation, in addition to reelin gene expression regulation by CpG promoter methylation, contributes to total reelin in adult mouse brain. Another possibility is that testosterone is signalling to neuronal maintenance, leading cells with specific methylation patterns to survive. In adult male mice, testosterone upregulates hippocampal neurogenesis via cell survival, although few studies compared possible differences between males and females [43]. Additionally, a key finding showed that absence of circulating testosterone in castrated rats leads to reduction of dendritic spines density of somatosensory cortical pyramidal neurons, while administration of exogenous testosterone brought this low density to normal levels [44]. Further experimental approaches are needed to provide more evidences to support these hypotheses.

## 5. Conclusions

In summary, we conclude that flutamide could be administered to male mice to bring plasmatic testosterone to low levels, which in turn lead to demethylation of* Reln* promoter cytosine residue. A more direct analysis of* reelin* CpG methylation would involve genomic sequencing, and a fine mapping of* Reln* promoter methylation pattern upon flutamide treatment will be key in understanding reelin gene expression in mouse brain. Our study contributes to the emerging insight that androgenic hormones can modulate cerebellar reelin expression.
